# Social interactions in dementia: perceptions of current situation and opportunities

**DOI:** 10.1186/s12877-025-05850-8

**Published:** 2025-03-27

**Authors:** Hanna L. Knecht, Francisca S. Rodriguez

**Affiliations:** German Center for Neurodegenerative Diseases (DZNE), RG Psychosocial Epidemiology & Public Health, Ellernholzstr. 1-2, 17489 Greifswald, Germany

**Keywords:** Dementia, Alzheimer’s, Social integration, Social medicine, Social health, Non-pharmacological, Psychosocial

## Abstract

**Background:**

Social interactions can have a beneficial effect on people with dementia (PWD). However, little is known about the details of social interactions in dementia, information that is useful for planning social interventions. The aim of our study was to gain a deeper insight, provided by people in dementia care, into (i) the characteristics of social interactions of PWD, (ii) which social interactions are considered as particularly important, (iii) how important social interactions are perceived, and (iv) what is perceived to increase social interactions among PWD.

**Methods:**

Responses of 501 people in dementia care (mostly family and professional caregivers; more than three-quarters female; average age 53.5 years) provided in a structured, quantitative survey were included in this analysis. Descriptive analyses were conducted.

**Results:**

The majority of PWDs’ social interactions take place in their home or day care and arise from support services as well as organized activities. More than half of the participants perceived singing, telling life stories, or moving together (e.g., walking, dancing) as valuable social interactions that PWD benefit from in many ways. Positive effects (e.g., aggression, depression, positive emotions, activation) are mainly perceived for social interactions such as laughing together, touch, hugging, simply being together, or being involved in daily activities. Overall, participants rated social interactions as rather important for the course of the symptoms. A fixed social contact besides the primary caregiver and offers from the community, associations, and religious institutions are considered rather important in increasing the social interactions of PWD.

**Conclusions:**

Simple social interactions of PWD such as laughing, eating or singing together that can be realized in the home environment and in the context of organized caregiving activities are perceived most valuable. Enhancing social interactions, especially beyond the primary caregiving context, may be valuable for those living with dementia.

**Supplementary information:**

The online version contains supplementary material available at 10.1186/s12877-025-05850-8.

## Background

Advanced age is usually accompanied by an increased risk of disease, which especially applies to neurodegenerative diseases such as dementia [1]. Worldwide, ageing societies are leading to rising numbers in people with dementia (PWD) that are expected to reach 115.4 million by 2050 [2]. This development is accompanied by an increased burden being put on different care settings, but most greatly on informal caregivers such as family members that usually take on the care of PWD [3].

Currently, there is no prevention or cure for dementia. However, numerous scientific steps towards researching new methods, especially in the non-pharmacological field, have already been taken. Thereupon, psychosocial aspects have been ascribed a high potential [4]. Research shows that high-quality social relationships, interacting with close contacts, and social integration into society have positive effects on both health in old age and cognitive performance [5–7]. This can, to some extent, be explained by social engagement enhancing the resilience to neuropathology in dementia [8] and by social interactions stimulating cognitive processes, which delay cognitive decline [9]. Moreover, positive effects on dementia symptoms have been observed in the presence of a social support network, which refers to the totality of all supportive social contacts [10]. In addition to the positive influences on cognitive health, large social networks and being socially active were associated with a higher quality of life in PWD in nursing homes amongst others [2, 11]. Explanations for these effects can be the social environment stimulating cognitive processes, reducing psychosocial stress, and positively influencing health-promoting behavior [12].

Although it is known that PWD benefit from social engagement, little is known about the characteristics of social interactions in dementia, especially in the context of Germany. Since this knowledge would form a valuable basis for the development of social interventions that could improve the life of PWD, this study aims to take an important step towards determining (i) the characteristics of social interactions of PWD, (ii) which social interactions are considered as particularly important, (iii) how important social interactions are perceived, and (iv) what is perceived to increase social interactions among PWD.

## Methodological approach

### Sample

A structured, quantitative survey was conducted with (i) PWD and (ii) people in dementia care (e.g., caregivers/ spouses/ partners in and outside the home, staff and coordinators in dementia networks, volunteers and full-time employees in facilities for PWD, and other people, who regularly deal with PWD).

The study was reviewed and approved by the Ethics Committee of the University Medical Center Greifswald (No. B017/23) and was conducted in accordance with the Declaration of Helsinki. Participants were recruited throughout Germany by disseminating the information about the study via email, newsletter, flyer, and social media. In addition to dementia networks, self-help and interest groups, professional associations as well as dementia advocates were contacted. Institutions, networks, and associations that are geared towards people from socially disadvantaged groups and people with a migration background were further contacted via email. General inclusion criteria for participation were (i) a minimum age of 18 years, (ii) the ability to consent (i.e., not in delirium or impaired consciousness), (iii) sufficient vision, and (iv) active involvement in dementia care. The approach in recruitment allowed us to gather information on institutionalized care settings and on dementia care taking place at home.

The survey took place in the period from Aug 15, 2023 to Feb 15, 2024 and was conducted using three formats between which the participants could freely choose: Either an online survey (95.6% of the participants), paper questionnaire (3.2% of the participants), or interview (at home or by phone, 1.2% of the participants). Each format contained the same questions.

### The questionnaire

As the aim of the study was to obtain an overall overview of the situation of PWD and many participants reported on several PWD that they were working with, it was not possible to use standardized assessments for social integration of PWD. Based on scientific literature and already published surveys on the topic of dementia, we also realized that existing questionnaires did not assess social interactions of PWD in enough detail. Accordingly, we created a structured questionnaire based on themes in published studies, an internal brainstorming session, and input from five external experts. The first version of the questionnaire was revised intensively internally as well as by five experts in the field of dementia regarding comprehensibility, duration, and completeness. The experts read the questionnaire, commented on different aspects of the questionnaire, and made suggestions for improvement, which were incorporated in the final version of the questionnaire. Since some questions were aimed exclusively at those in dementia care and since additional information was requested from PWD, two questionnaire versions were created, suitable either for those in dementia care or PWD. In the following, it is indicated whether solely PWD, only participants without dementia or all participants answered the questions. Finally, participants were not obliged to answer any of the questions, resulting in different numbers of responses per question.

The questionnaire first assessed personal information (age, gender, education (e.g., high school diploma or higher education), income, role in dementia care, urban/rural setting, and cultural identity).

Details on the questions to each section on social interactions and the respective answer formats are shown in the supplementary file, Table S1. To describe the characteristics of social interactions, we asked participants to rank given activities according to their importance in leading to social interaction and posed single choice questions on (i) who initiates social interactions for PWD and on (ii) where most of the social interactions take place as well as multiple choice questions on (I) which social interactions are most valuable in daily care and on (II) what the most common reasons for the reduction of the social network are. A multiple choice question posed at participants without dementia further inquired which social interactions help activate PWD at advanced stages and a single choice question inquired with which person (group) the relationship breaks off most often. In another single choice question, all participants were asked about whether new social relationships were developing after the dementia diagnosis. Moreover, we inquired, using a Likert scale, how frequently PWD (i) actively conduct a conversation with others, (ii) experience touch (e.g., caressing hand), and (iii) have sex. Additionally, only PWD were asked, using a Likert scale, which out of ten social skills they still have despite their dementia.

To assess which social interactions are perceived as having a positive impact, we asked participants in multiple choice questions which social interactions (i) trigger positive emotions and (ii) make PWD feel needed. In a free text field, they were encouraged to indicate which social interactions promote the self-esteem of PWD. Participants without dementia were further asked multiple choice questions about which social interactions (i) provide safety and security, (ii) calm aggressive behavior, and (iii) influence depression (for details see supplementary file, Table S1.).

To determine perceptions on the importance of social interactions on symptoms, participants where asked, using a Likert scale, whether they agreed that (i) regular social interactions preserve cognitive performance, (ii) involving PWD in activities of daily living reduces dementia symptoms, and that (iii) socially integrated PWD have a high quality of life (for details see supplementary file, Table S1.).

To assess methods to increase social interactions among PWD, participants were asked, using a Likert scale, how important it is for the course of the disease that (i) social interactions are actively established, (ii) a fixed social professional contact person for PWD exists, (iii) understanding for dementia symptoms is shown, (iv) offers from the community, associations and religious institutions for PWD are available, and that (v) there is an exchange between relatives and professionals. We further inquired, using a Likert scale, whether participants agreed that technology-supported interventions (e.g., apps) can support social interactions. A similar question but concerning robots (e.g., animal, human) was posed solely to participants without dementia (for details see supplementary file, Table S1.).

### Analysis

To be able to calculate frequencies of free texts provided by participants, in a first step, any such information (i.e., answers to the option “other” in the multiple choice questions) was sorted into categories by two independent researchers. In consensus meetings, discrepancies in the category assignment were resolved. Accordingly, frequencies of these additional answer categories could be calculated.

Subsequently, the frequencies and mean values for all responses to all questions were calculated. We also made overall and pairwise comparisons for all the questions. Data analysis was conducted using the statistical software STATA, version 17.

## Results

Participants were on average 53.5 years old (SD = 12.4, range 19–91). Further details on the sample are described in Table 1.

**Table 1:** Sociodemographic characteristics of participants; *n* = 501

Characteristic	*n*	%
Gender		
Female	418	85.8
Male	69	14.2
Highest educational level		
High school diploma or higher education	303	64.7
Middle school or lower education	165	35.3
Living environment		
Urban	259	53.1
Rural	229	46.9
Income		
High	48	9.9
Middle	398	82.4
Low	37	7.7
Cultural identity other than German		
Yes	49	10.0
No	439	90.0
Other cultural identity		
European	23	50.0
Asian	8	17.4
Middle Eastern	7	15.2
Role in dementia care		
Family caregiver	182	38.3
Professional caregiver	145	30.5
Others	135	28.4
Employee in counselling PWD	99	20.8
Therapists	25	5.3
In other ways involved in dementia care	11	2.3
Person with dementia	14	2.9

Notes: *n*, number of participants; PWD, people with dementia

### Characteristics of social interactions

Participants were asked to assign ranks (with 1 being the most important) to what activities are most likely to result in social interactions for PWD. As most important, indicated by a low mean rank, were perceived to be organized activities (M = 2.9, SD = 2.0) and support services (e.g., home care; M = 3.1, SD = 1.9), followed by encounters in the neighborhood (M = 3.7, SD = 1.9) and leisure activities (M = 3.8, SD = 1.9). Perceived less important were doctor appointments (M = 4.3, SD = 1.9), procurement of food and medication (M = 4.9, SD = 1.9), religious events (M = 5.7, SD = 2.0) and coincidental interactions (M = 6.9, SD = 1.7).

Details on the frequencies of the participants’ responses on details of social interactions are shown in Table 2. As to the person normally initiating PWDs’ social interactions, participants indicated PWDs’ social contacts as well as doctors and therapists. Moreover, home and day care were places mainly selected for where most of PWDs’ social interactions take place (see Table 2). Regarding the frequency of interactions, participants agreed that PWD sometimes conduct active conversations with others (M = 2.8, SD = 0.8) and experience touch (M = 3.2, SD = 0.9), but seldom have sexual intercourse (M = 1.9, SD = 0.7). As to the most valuable interactions in daily care, participants most commonly selected smiles, touch, telling ones’ life story, simply looking at each other, singing, and holding conversations (see Table 2). Regarding activating PWD in advanced stages, participants without dementia agreed on singing or humming, touch (e.g., stroking), moving together (e.g., dancing), holding hands, and using familiar objects (see Table 2). Furthermore, as to the relationships breaking off after the diagnosis, the greater part of participants without dementia responded that the relationships to friends and acquaintances is mainly affected (see Table 2). As for the reasons of the reduction of the social network, most of the participants named the decrease in the PWDs’ ability to maintain social contacts, others being overwhelmed by dementia symptoms, and an increased need for support (see Table 2). Moreover, new social contacts can be made after a dementia diagnosis, as the majority of participants agreed upon (see Table 2).

**Fig. 1:**
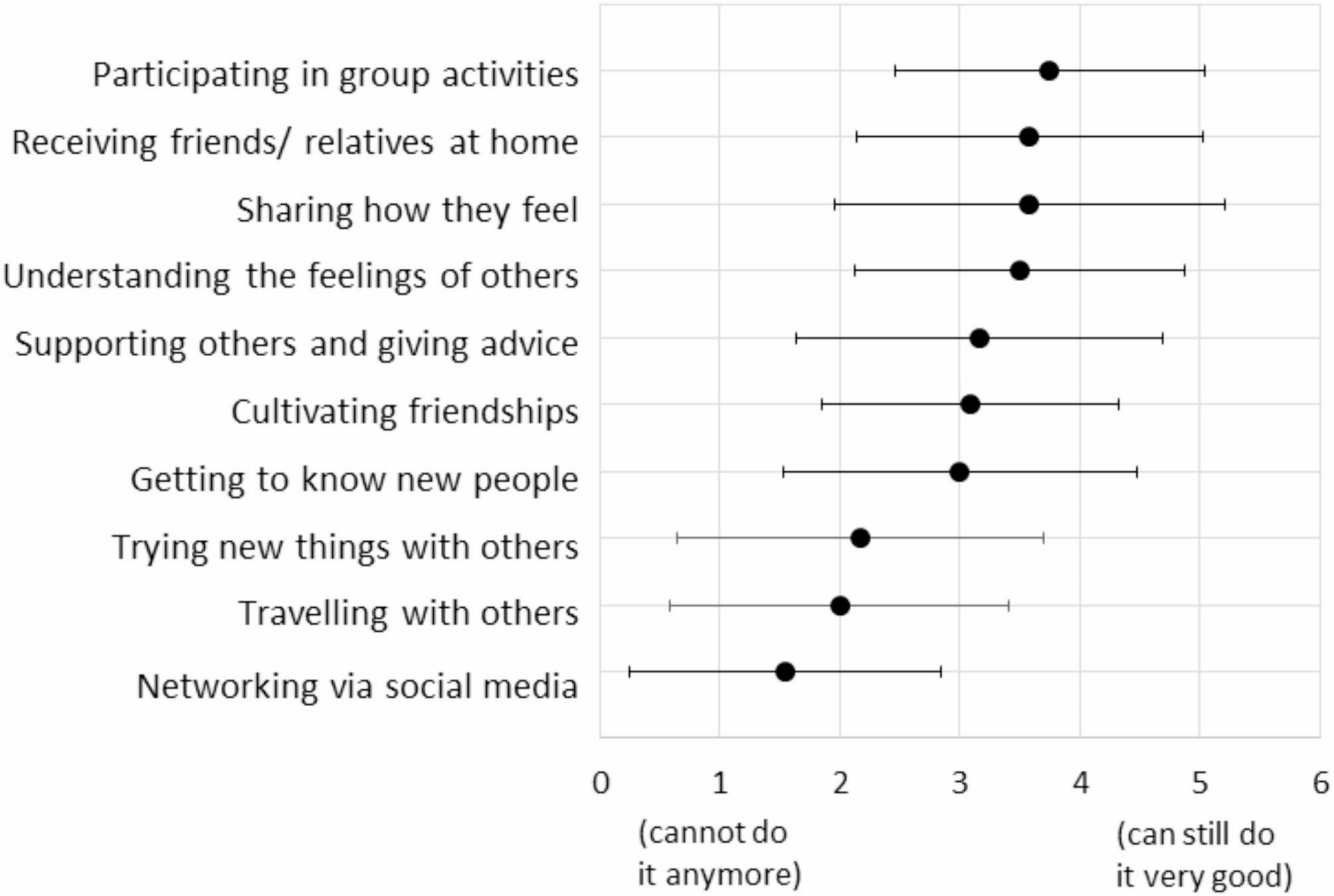
Average answers from solely PWD on the question what they still can do; responses on a Likert scale from cannot do it anymore (1) to can still do it very good (5). Error bars indicate standard deviations

Regarding the remaining social skills of PWD, participants with dementia indicated that they are still able to participate in group activities, to receive friends/relatives at home, to share how they feel, and to understand the feelings of others (see Fig. 1). On the other hand, they reported not being able to network via social media and travel with others (see Fig. 1).

### Social interactions that have a positive impact

Details on the social interactions that the participants named to have a positive impact are also listed in Table 2. Most participants reported that to trigger positive emotions in PWD, simply being together, laughing together, and undertaking joint activities (e.g., singing, traveling, playing or alike) are perceived as useful. To achieve feelings of safety and security, most participants without dementia selected eating together, hugging, and singing (see Table 2). To promote the self-esteem of PWD, most participants agreed on carrying out meaningful activities (e.g., cooking together), experiencing competence (e.g., being asked for advice), and engagement in social activities (see Table 2). Further, being involved in everyday activities or activities that they used to do (e.g., watering the garden as a former hobby gardener) and “being asked for help” enables PWD to experience the feeling of being needed (see Table 2).

To calm aggressive behavior, most participants without dementia suggested “staying calm”, signaling that the PWDs’ feelings are taken seriously (e.g., validation), and touch (e.g., stroking a hand; see Table 2) as most useful interactions. To improve depressive behavior, most participants without dementia selected involvement in daily activities, signaling that feelings and needs are taken seriously, and touch (e.g., stroking a hand; see Table 2).

**Table 2:** Answers chosen by participants on questions regarding the characteristics of social interactions and on what social interactions are beneficial for PWD; *n* = 501

Question	Answer	% (*n*)
Characteristics of social interactions		
People normally initiating social interactions §	Social contacts of PWD	78.2 (358)
	Doctors and therapists	8.1 (37)
	PWD themselves	5.9 (27)
Place where most social interactions take place §	At home	59.6 (273)
	In day care	20.7 (95)
	In the neighborhood	6.6 (30)
	At social meeting places	6.3 (29)
Most valuable interactions in daily care §	Smiles	82.6 (394)
	Touch	78.8 (376)
	Telling ones’ life story	68.6 (327)
	Simply looking at each other	63.5 (303)
	Singing	60.6 (289)
	Conversations	60.2 (287)
	Talking about problems or fears	32.7 (156)
	Talking about wishes or the future	26.4 (126)
Activating PWD in advanced stages $	Singing or humming	76.8 (274)
	Touch (e.g., stroking)	73.4 (262)
	Moving together (e.g., dancing)	68.6 (245)
	Holding hands	63.3 (226)
	Using familiar objects	52.9 (189)
	Mirroring gestures, sounds or posture	34.5 (123)
	Stimulating senses	2.5 (9)
Relationships that break off most $	Friends and acquaintances	48.9 (184)
	Contacts from work/school	26.6 (100)
	Contacts from social media	9.3 (35)
	Neighbors	6.9 (26)
	Family members	2.4 (9)
	Service providers	1.9 (9)
Reasons for the reduction of the social network §	Decrease in ability to maintain social contacts	85.5 (349)
	Others overwhelmed by diagnosis/symptoms	83.8 (342)
	Increase in the need for support	67.4 (275)
	Decline in mobility	66.7 (272)
	By PWD due to shame/feeling of worthlessness	62.5 (255)
	Lack of support	60.3 (246)
	Others not informed about disease	59.8 (244)
	Others withdraw due to lack of time or unwillingness to invest time	56.9 (232)
	Death of social contacts	51.0 (208)
	Moving into nursing home	45.6 (186)
	Low income	13.2 (54)
	Non-dementia-related impairments (e.g., poor hearing)	1.2 (6)
	Excessive demands in social contacts	1.2 (6)
New social contacts being made after dementia diagnosis §	Yes	48.8 (185)
	No	37.7 (144)
	Rarely	4.5 (17)
Social interactions that have a positive impact		
Triggering positive emotions §	Being together	85.3 (324)
	Laughing together	80.0 (304)
	Joint activities (e.g., singing, travelling, playing games)	79.7 (303)
	Participating in daily activities	64.5 (245)
	Cuddling, physical closeness or sex	50.5 (192)
	Conversations	50.3 (191)
	Biography work	43.2 (164)
	Visiting friends	30.0 (114)
	Performing personal hygiene	16.1 (61)
	Interactions in nature with/without animals	1.0 (5)
Providing safety and security $	Eating together	67.0 (246)
	Hugging	66.5 (244)
	Singing	62.9 (231)
	Being active together	55.9 (205)
	Everyday support	52.0 (191)
	Conversations	51.2 (188)
	Sitting together (e.g., watching movies)	45.5 (167)
	Playing together	42.8 (157)
	Performing personal hygiene	12.5 (46)
	Family moments	1.2 (6)
Promoting self-esteem §	Carrying out meaningful activities (e.g., cooking together)	50.6 (125)
	Experiencing competence (e.g., being asked for advice)	33.3 (83)
	Engagement in social activities	21.6 (54)
	Conveying appreciation	18.2 (45)
Feeling needed §	Being involved in daily activities	87.6 (331)
	Assisting in activities they used to perform	68.0 (257)
	Being asked for help	65.9 (249)
	Being asked for advice/own opinion	54.8 (207)
	When their motivation and effort is valued	47.1 (179)
	Receiving invitations to social activities	47.4 (179)
	Receiving visits	37.3 (141)
Calm aggressive behavior $	Staying calm	82.0 (292)
	Signaling that their feelings are taken seriously	75.6 (269)
	Touch	54.8 (195)
	Distraction	52.5 (187)
	Figuring out underlying reasons	23.0 (82)
	Leaving the situation	20.5 (73)
	Individual strategies (e.g., saying stop)	2.8 (10)
Influence depressive symptoms $	Being involved in daily activities	72.3 (258)
	Signaling that their feelings are taken seriously	68.1 (243)
	Touch	67.2 (240)
	Being involved in interest-based activities	52.7 (188)
	Distraction	40.3 (144)
	Figuring out underlying reasons	34.2 (122)

Notes: §, question for all participants; $, question solely for participants without dementia; n, number of participants; PWD, people with dementia

### Perceptions on the importance of social interactions

**Fig. 2:**
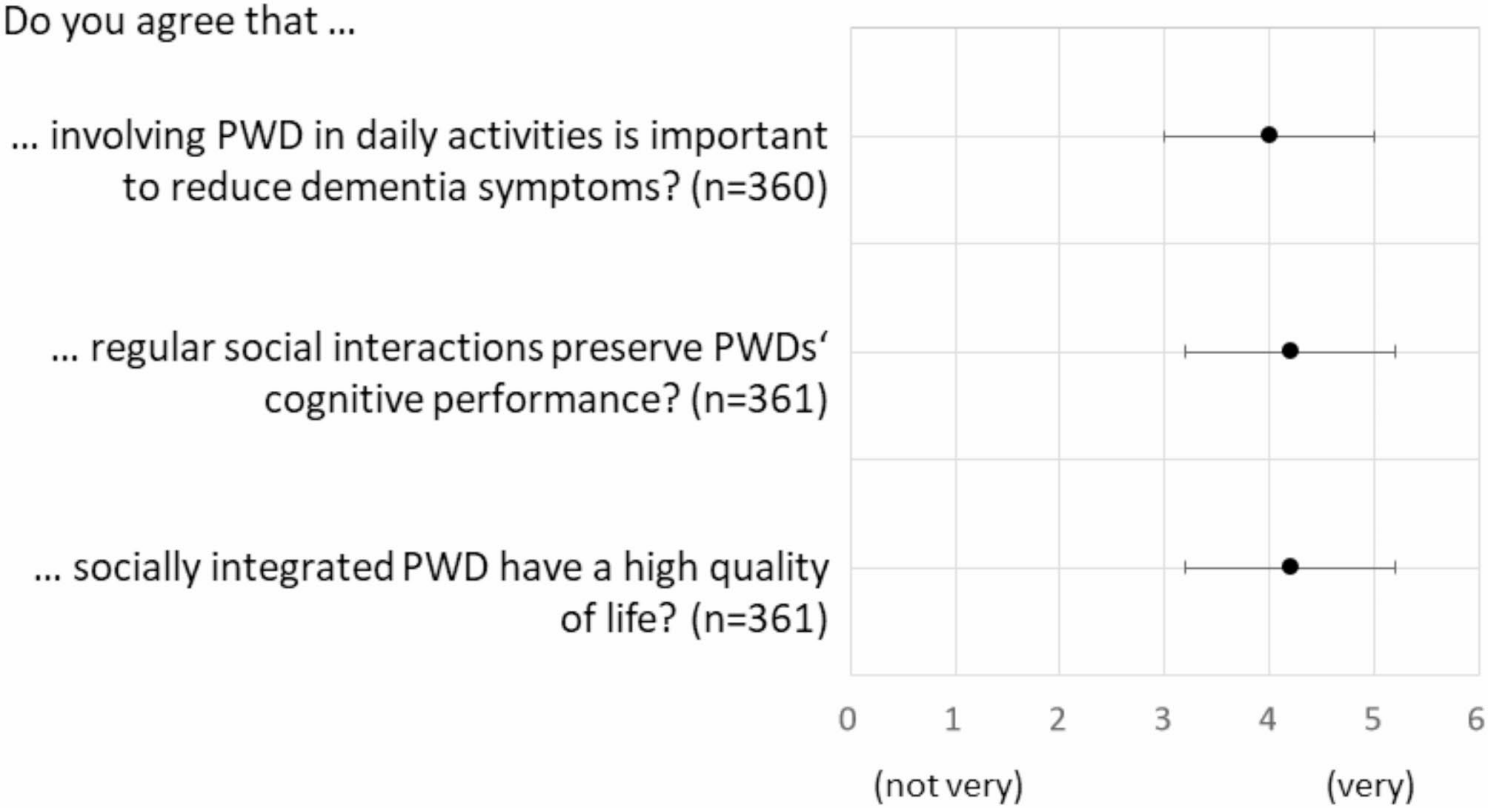
Average answers from all participants on a Likert scale from agreeing not very (1) to very (5) on the importance of social interactions. Error bars indicate standard deviations

Results regarding the importance of social interactions are shown in Fig. 2. To reduce dementia symptoms, respondents ‘tended’ (not ‘strongly’) to agree that involving PWD in daily activities is important. Moreover, they ‘tended’ (not ‘strongly’) to agree with the idea that regular social interactions preserve PWDs’ cognitive performance (see Fig. 2). On average, participants further ‘tended’ (not ‘strongly’) to agree that socially integrated PWD have a high quality of life (see Fig. 2).

### Perceptions on methods to increase social interactions

**Fig. 3:**
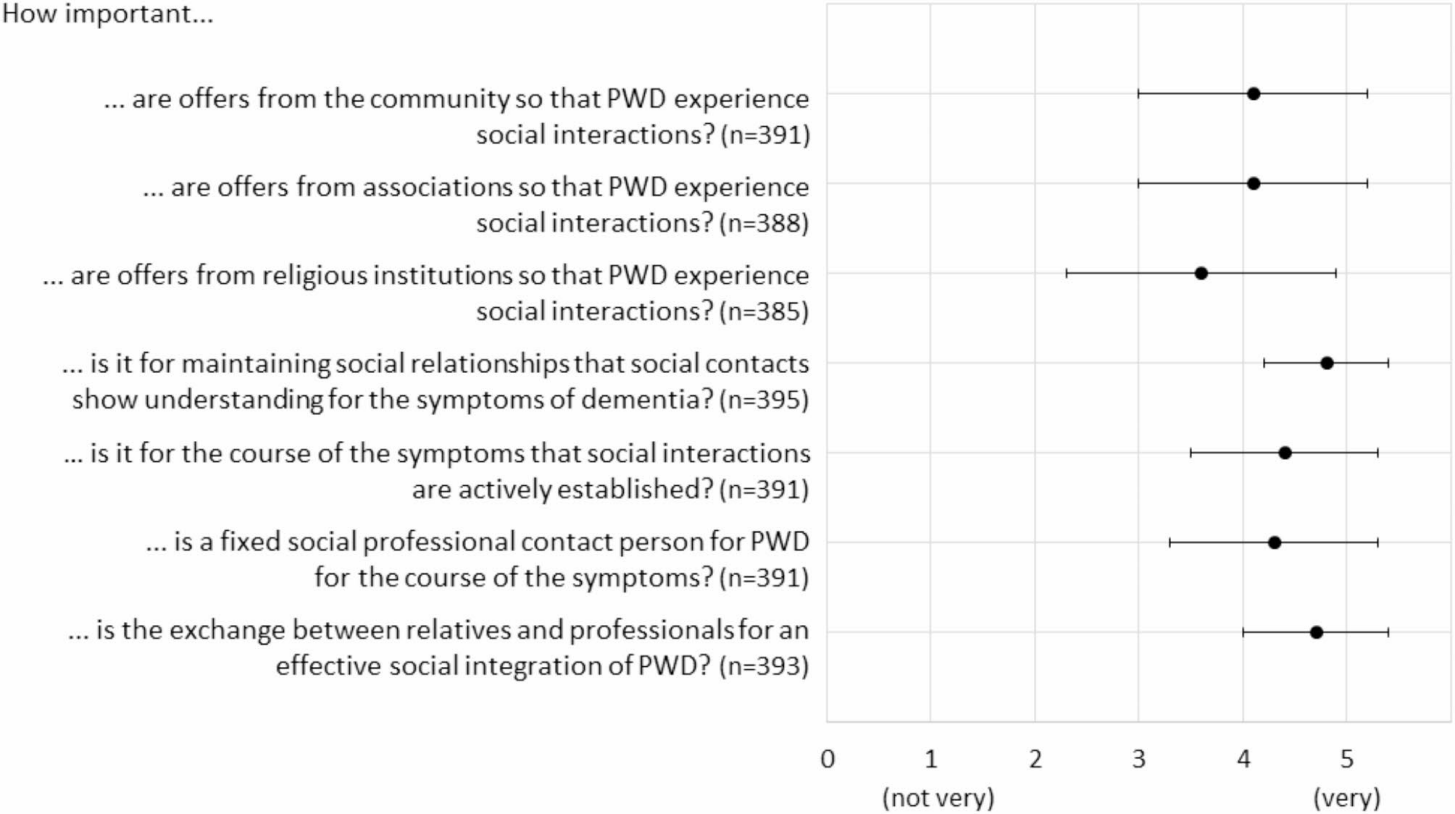
Average answers from all participants on a Likert scale from not important (1) to very important (5) on what can improve social interactions among PWD. Error bars indicate standard deviations

Participants’ average responses to the questions are shown in Fig. 3. The active initiation of social interactions for PWD, a fixed professional social contact for PWD in addition to doctors, nurses, family and friends, and offers from the community, associations, and religious institutions are perceived to be ‘rather’ (not ‘very’) important. Showing an understanding for dementia symptoms as well as an exchange between relatives and professionals is perceived as ‘very’ important (see Fig. 3). Regarding the use of technology, participants with and without dementia ‘tended’ (not ‘strongly’) to disagree that technology-supported interventions (e.g., apps) could support the social interaction of PWD (M = 2.3, SD = 1.1), whereas the idea that robots (e.g., in the form of an animal or human) could support their social interaction was neither agreed to nor rejected by participants without dementia (M = 2.5, SD = 1.2).

### Differences between participants with different roles in dementia care

Differences in responses between PWD, family caregivers, professional caregivers, and participants otherwise connected to dementia care are shown in the supplementary file, Tables S2 and S3. It is noticeable that most statistically significant differences are found between family caregivers and professional caregivers/ others. For example, family caregivers describe a smaller relevance to talking about fears and wishes, touching, singing, mirroring gestures, joint activities, carrying out meaningful or interest-based activities, and signaling that they take PWD’s emotions serious. Professional caregivers, on the other hand, perceived a greater relevance of participating in daily activities, organized activities, and religious activities. Even though only a relatively small number of PWD participated, their responses suggest that, compared to the other participants, they see telling life stories and offers in the community or by associations as less important, but emphasize the relevance of leisure activities and spontaneous encounters.

## Discussion

Our findings indicate that the majority of PWDs’ social interactions take place in their home or day care and arise from support services and organized activities. A decreasing ability to maintain social contacts, feelings of shame, and social contacts being overwhelmed by dementia symptoms are important reasons for loosing social contacts, most frequently friends. Laughing, singing, telling one’s life story, or moving together (e.g., walking, dancing) are perceived as valuable social interactions, while positive effects are achieved through interactions such as touch, hugging, simply being together, being involved in daily activities or engaging in any other type of activity together. Overall, participants rated social interactions as important for the course of the symptoms. Arranging a fixed social contact besides the primary caregiver and offers from the community, associations, and religious institutions are considered helpful in increasing the social interactions of PWD.

Findings indicating the importance of familiar environments for PWDs’ social interactions to take place are in line with the current literature. It was shown that homelike settings best promote interactions, which could be explained by known objects serving as stimulators and PWD feeling comfortable to interact with others in this setting [13]. Moreover, PWD are generally described as individuals dependent on support from their social contacts – mainly primary caregivers – for instance regarding daily life, but especially for social wellbeing [3]. This may indicate the importance of focusing on how to support primary caregivers in enabling social interactions for PWD in their respective homes. Previous studies further reported that a great percentage of interactions occur during planned activities [14], underlining that the initiation of interactions depends on organized activities. For this reason, we highlight the necessity of strengthening organized activities, particularly those by communities, religious institutions, and associations that have been ascribed the potential to meet those needs [15]. This could also help to prevent a loss of social interactions after a dementia diagnosis [16]. Loss of social contacts occurs, as suggested by our findings and the wider literature, because PWD feel stigmatized and actively withdraw from society to circumvent negative experiences [17]. Further, social contacts avoid contact with PWD because they are overwhelmed by dementia symptoms such as noise-making [18]. This points out the urgent need to achieve an understanding of dementia in the society as well as the need to improve PWDs’ own acceptance. Since friends have been identified as being the contact to most often be lost [19], they should be considered in this context.

Our results highlight several non-verbal means of social interaction (e.g., smiling) that are of great value, which might be explained by the fact that PWD eventually lose their ability to communicate verbally and naturally revert to facial and other non-verbal expressions that still allow feelings to be conveyed [20]. Especially touch is further associated with beneficial effects such as reducing behavioral symptoms [21], triggering feelings of emotional connectedness, and stimulating physical reactions [22], amongst others. Being considered valuable in daily care, for activating PWD in advanced stages, and for the course of the disease, particularly touch could be regarded as an essential social interaction in dementia care.

Our survey highlighted several social interactions to positively impact PWD, especially the involvement in both everyday and meaningful activities. This resonates with studies describing that PWD experience joy in numerous social activities jointly executed [3] and that PWD feel a sense of purpose when helping others with everyday chores [23]. In that context, the importance of interactions conveying that PWD are still meaningful individuals was further supported by our participants. Recent studies underlined that inquiring about PWDs’ opinion and that encouraging them to fulfill their new roles’ potential by helping others – representing a reciprocal relationship – results in the feeling of being needed [24]. A similar effect can be seen in PWDs’ aggressive and depressive behavior being adequately met by contacts expressing that their feelings are taken seriously. This may suggest enabling PWD to engage and thrive in meaningful everyday activities.

Besides its’ significance in triggering positive emotions, participation in social activities (including meaningful everyday activities) was, in this study, considered important for reducing dementia symptoms as well as in preserving cognitive performance. This is supported by previous research that has shown that social participation can reduce cognitive deterioration, for instance, by stimulating environments increasing cognitive processes and buffering stress [25], and that it lessens neuropsychiatric dementia symptoms such as agitation, which could express unmet social needs [26]. Therefore, it seems imperative that remaining socially active should be a common goal in dementia care that all PWDs’ social contacts should be invested in achieving.

Limitations of this study are specified in the following. First, our findings are based on subjective experiences of people in dementia care and should only be interpreted as such. Second, the study does not allow us to infer differences regarding the type or stage of dementia. This will have to be investigated in more detail in future studies. Furthermore, even though we offered the survey in different formats to allow as many people as possible to participate, we cannot exclude a selection bias. Moreover, despite our efforts, PWD and their lived experiences – considered most important in this context – are highly underrepresented (3%). Generalizability of the findings is therefore constrained.

Even though we believe that the questionnaire that was used provides valuable information, it is important to acknowledge that it is not a validated instrument. However, since comments from experts based on revision of the questionnaire draft have been incorporated, limitations have been kept to a minimum. Only a separate study specifically with PWD may conclude to what extent the questions on valuable social interactions represent the level of social integration. Possible biases that may have occurred by giving participants a selection of answers were reduced by always providing them with the opportunity to add their own responses. However, further research using focus group discussions or semi-structured interviews would be beneficial to gain a deeper understanding, as would be including information about the dementia stage and exploring the types of social interaction in detail.

## Conclusion

Our findings provide an insight into the PWDs’ social interactions by informing about its’ characteristics as well as about valuable types of social interactions and stakeholder’s perceptions on their relevance and ways of enhancing them. Highlighted should be the importance of organized and meaningful everyday activities as well as non-verbal interactions such as touch. Even though additional efforts to enhance social interactions with PWD may be perceived as another burden to caregivers, the benefits on neuropsychiatric symptoms, wellbeing, and cognitive decline may outweigh the burden. More research is necessary to evaluate that.

## Electronic supplementary material

Below is the link to the electronic supplementary material.

Supplementary Material 1

## Data Availability

The datasets generated and/or analyzed during the current study are not publicly available due to restrictions in the informed consent and are available from the corresponding author upon request only if eligible under the local data protection laws.

## Abbreviations

PWD: People with dementia
