# Supplementary material for: Social interactions in dementia: perceptions of current situation and opportunities
Source: BMC Geriatr. 2025 Mar 27;25:202. doi: 10.1186/s12877-025-05850-8 (PMC11951574; doi:10.1186/s12877-025-05850-8)
Supplement: Supplementary file 1 — Supplementary Material 1 [file 12877_2025_5850_MOESM1_ESM.docx]

**Supplementary file**

**to the manuscript “Social interactions in dementia: Perceptions of current situation and opportunities”**

**Supplementary table S1.** Topics, question contents, and the respective answer formats

| Topic | Questions | Response method |
| --- | --- | --- |
| Characteristics of social interaction | “How important are the following activities in leading to social interactions?”   - Organized activities - Support services (e.g. home care) - Encounters in the neighborhood - Leisure activities - Doctor’s appointment - Buying food and medicine - Religious events - Coincidentally | Rank: most important (1) to least important (8) |
|  | “Who initiates social interactions for PWD?” | Single choice:   - PWD - Social contacts of PWD - Doctors, therapists - Coincidentally - Others (comment) |
|  | “Where do most social interactions of PWD take place?” | Single choice:   - At home - In the neighborhood - In places of the community (e.g. park) - In religious institutions - At social meeting places - In day care - At sports associations - At self-help groups - At the doctor‘s and therapy practice - In shopping settings - On social media - Elsewhere (comment) |
|  | “How frequently do PWD …”   - Conduct active conversations? - Experience touch? - Have sex? | Likert scale: never (1) to always (5) |
|  | “Which social interactions in daily care are perceived most valuable?” | Multiple choice:   - Conversations - Touch - Smiles - Simply looking at each other - Singing - Telling ones’ life story - Talking about problems or fears - Talking about wishes or the future - Others (comment) |
|  | Participants without dementia: “Which social interactions help activate PWD in an advanced stage?” | Multiple choice:   - Touch (e.g. stroking) - Singing or humming - Moving together (e.g. dancing) - Holding hands - Mirroring gestures, sounds or posture - Using familiar objects - Others (comment) |
|  | Participants without dementia: “The relationship to which person/ group breaks off most?” | Single choice:   - Partner/ spouse - Family members - Friends and acquaintances - Neighbors - Professional caregiver - Contacts from activities for PWD - Doctors - Therapists - Contacts from self-help groups - Contacts from work/school - Contacts from social media - Service providers - Volunteers - Others (comment) |
|  | “What are the most common reasons for the reduction of the social network?” | Multiple choice:   - Decrease in ability to maintain social contacts - Others overwhelmed by diagnosis/ symptoms - Increase in the need for support - Decline in mobility - By PWD due to shame/ feeling of worthlessness - Lack of support - Others not informed about disease - Others withdraw due to lack of time or unwillingness to invest time - Death of social contacts - Moving into nursing home - Low income - Others (comment) |
|  | “Do new social relationships develop after the dementia diagnosis?” | Single choice:   - Yes - No - Comment |
|  | PWD: “Despite of my dementia, I can still …”   - Receive friends/relatives at home - Participate in group activities - Support others and give advice - Get to know new people - Share how I feel - Understand the feelings of others - Cultivate friendships - Network via social media - Travel with others - Try new things with others | Likert scale: cannot do it anymore (1) to can still do it very good (5) |
| Social interactions that have a positive impact | “Which social interactions trigger positive emotions?” | Multiple choice:   - Cuddling, physical closeness, or sex - Conversations - Being together - Participating in daily activities - Laughing together - Joint activities (e.g. singing, travelling, playing games) - Biography work - Performing personal hygiene - Visiting friends - Others (comment) |
|  | “Which social interactions make PWD feel needed?” | Multiple choice:   - Being involved in daily activities - Being asked for advice/own opinion - Being asked for help - When their motivation and effort is valued - Assisting in activities they used to perform - Receiving invitations to social activities - Receiving visits - Others (comment) |
|  | “Which social interactions promote the self-esteem of PWD?” | Free text field |
|  | Participants without dementia: “Which social interactions provide safety and security?” | Multiple choice:   - Everyday support - Performing personal hygiene - Conversations - Hugging - Playing together - Singing - Eating together - Sitting together (e.g. watching movies) - Being active together - Others (comment) |
|  | Participants without dementia: “Which social interactions calm aggressive behavior?” | Multiple choice:   - Signaling that their feelings are taken seriously - Distraction - Figuring out underlying reasons - Staying calm - Touch - Leaving the situation - Others (comment) |
|  | Participants without dementia: “Which social interactions influence depression?” | Multiple choice:   - Signaling that their feelings are taken seriously - Distraction - Figuring out underlying reasons - Being involved in daily activities - Touch - Being involved in interest-based activities - Others (comment) |
| Perceptions on the importance of social interactions | “Do you agree that …”   - Regular social interactions preserve cognitive performance - Involving PWD in activities of daily living reduces dementia symptoms - Socially integrated PWD have a high quality of life | Likert scale: not very (1) to strongly agreeing (5) |
| Perceptions on methods to increase social interactions | “How important is it for the course of the disease that …”   - Social interactions are actively established - A fixed social professional contact person for PWD exists - Understanding for dementia symptoms is shown - Offers from community, associations, and religious institutions are available - There is an exchange between relatives and professionals | Likert scale: not very (1) to very important (5) |
|  | “Do you agree that technology-supported interventions can support social interactions?” | Likert scale: not very (1) to strongly agreeing (5) |
|  | Participants without dementia: “Do you agree that robots can support social interactions?” | Likert scale: not very (1) to strongly agreeing (5) |

Notes: PWD, people with dementia.

**Supplementary table S2**. Differences in frequencies of answers between PWD, family caregiver (FC), professional caregivers (PC), and people otherwise connected to dementia care (others); n=476

| Question | Answer | Overall comparison | | | | | Pairwise comparison *,§ | | | | | |
| --- | --- | --- | --- | --- | --- | --- | --- | --- | --- | --- | --- | --- |
|  |  | PWD (n=14) | FC (n=182) | PC (n=145) | Others (n=135) |  | PWD - FC | PWD - PC | PWD - Others | FC - PC | FC - Others | PC - Others |
|  |  | % (n) | % (n) | % (n) | % (n) | X² (p) | X² (p) | X² (p) | X² (p) | X² (p) | X² (p) | X² (p) |
| **People initiating social interaction** |  |  |  |  |  |  |  |  |  |  |  |  |
| PWD |  | 30.8 (4) | 7.1 (12) | 6.7 (9) | 0.8 (1) | 35.690 (0.002) |  |  |  |  |  |  |
| Social contacts |  | 46.2 (6) | 79.4 (135) | 77.6 (104) | 79.7 (102) |  |  |  |  |  |  |  |
| Doctors/ therapists |  | 7.7 (1) | 7.1 (12) | 9.0 (12) | 8.6 (11) |  |  |  |  |  |  |  |
| **Social interactions taking place** |  |  |  |  |  |  |  |  |  |  |  |  |
| At home |  | 41.7 (5) | 60.8 (104) | 62.0 (85) | 54.4 (68) | 59.244 (0.003) |  |  |  |  |  |  |
| Neighborhood |  | 8.3 (1) | 5.3 (9) | 7.3 (10) | 7.2 (9) |  |  |  |  |  |  |  |
| Social meeting-points |  | 0.0 (0) | 2.9 (5) | 5.8 (8) | 12.0 (15) |  |  |  |  |  |  |  |
| Day care |  | 25.0 (3) | 22.8 (39) | 20.4 (28) | 20.0 (25) |  |  |  |  |  |  |  |
| **Valuable interactions in daily care** |  |  |  |  |  |  |  |  |  |  |  |  |
| Conversations | Yes | 50.0 (6) | 64.2 (113) | 62.3 (86) | 59.7 (77) | 1.394 (0.707) |  |  |  |  |  |  |
|  | No | 50.0 (6) | 35.8 (63) | 37.7 (52) | 40.3 (52) |  |  |  |  |  |  |  |
| Touch | Yes | 25.0 (3) | 82.4 (145) | 86.2 (119) | 79.1 (102) | 27.546 (<0.001) |  |  |  | 0.854 (0.355) | 0.532 (0.466) | 2.398 (0.121) |
|  | No | 75.0 (9) | 17.6 (31) | 13.8 (19) | 20.9 (27) |  |  |  |  |  |  |  |
| Smiles | Yes | 33.3 (4) | 85.8 (151) | 89.9 (124) | 83.0 (107) | 27.928 (<0.001) |  |  |  | 1.172 (0.279) | 0.464 (0.496) | 2.729 (0.99) |
|  | No | 66.7 (8) | 14.2 (25) | 10.1 (14) | 17.1 (22) |  |  |  |  |  |  |  |
| Looking at each other | Yes | 16.7 (2) | 62.5 (110) | 70.3 (97) | 69.0 (89) | 15.460 (0.001) |  |  |  | 2.089 (0.148) | 1.384 (0.239) | 0.053 (0.818) |
|  | No | 83.3 (10) | 37.5 (66) | 29.7 (41) | 31.0 (40) |  |  |  |  |  |  |  |
| Singing | Yes | 8.3 (1) | 54.6 (96) | 75.4 (104) | 63.6 (82) | 29.466 (<0.001) |  |  |  | 14.495 (<0.001) | 2.493 (0.114) | 4.390 (0.036) |
|  | No | 91.7 (11) | 45.5 (80) | 24.6 (34) | 36.4 (47) |  |  |  |  |  |  |  |
| Telling ones‘ life story | Yes | 41.7 (5) | 65.3 (115) | 72.5 (100) | 76.0 (98) | 8.973 (0.030) | 2.727 (0.099) | 4.986 (0.026) | 6.562 (0.010) | 1.818 (0.178) | 3.992 (0.046) | 0.428 (0.513) |
|  | No | 58.3 (7) | 34.7 (61) | 27.5 (38) | 24.0 (31) |  |  |  |  |  |  |  |
| Talking about problems or fears | Yes | 25.0 (3) | 25.0 (44) | 40.6 (56) | 38.8 (50) | 10.781 (0.013) |  |  |  | 8.650 (0.003) | 6.610 (0.010) | 0.092 (0.761) |
|  | No | 75.0 (9) | 75.0 (132) | 59.4 (82) | 61.2 (79) |  |  |  |  |  |  |  |
| Talking about wishes or the future | Yes | 8.3 (1) | 19.9 (35) | 33.3 (46) | 31.8 (41) | 10.937 (0.012) |  |  |  | 7.307 (0.007) | 5.631 (0.018) | 0.073 (0.787) |
|  | No | 91.7 (11) | 80.1 (141) | 66.7 (92) | 68.2 (88) |  |  |  |  |  |  |  |
| **Reasons for reduction of social network** |  |  |  |  |  |  |  |  |  |  |  |  |
| Others overwhelmed by diagnosis/ symptoms | Yes | 36.4 (4) | 84.6 (132) | 88.2 (105) | 87.7 (93) | 22.263 (<0.001) |  |  |  | 0.743 (0.389) | 0.507 (0.477) | 0.013 (0.908) |
|  | No | 63.6 (7) | 15.4 (24) | 11.8 (14) | 12.3 (13) |  |  |  |  |  |  |  |
| By people with dementia due to shame/ feeling of worthlessness | Yes | 27.3 (3) | 50.0 (78) | 73.1 (87) | 77.4 (82) | 32.111 (<0.001) |  |  |  | 15.021 (<0.001) | 19.870 (<0.001) | 0.542 (0.462) |
|  | No | 72.7 (8) | 50.0 (78) | 26.9 (32) | 22.6 (24) |  |  |  |  |  |  |  |
| Decrease in ability to maintain social contacts | Yes | 63.6 (7) | 89.7 (140) | 87.4 (104) | 83.0 (88) | 7.502 (0.058) |  |  |  |  |  |  |
|  | No | 36.4 (4) | 10.3 (16) | 12.6 (15) | 17.0 (18) |  |  |  |  |  |  |  |
| Increase in the need for support | Yes | 27.3 (3) | 73.7 (115) | 74.8 (89) | 60.4 (64) | 16.162 (0.001) |  |  |  | 0.041 (0.841) | 5.190 (0.023) | 5.352 (0.021) |
|  | No | 72.7 (8) | 26.3 (41) | 25.2 (30) | 39.6 (42) |  |  |  |  |  |  |  |
| Death of social contacts | Yes | 9.1 (1) | 43.6 (68) | 65.6 (78) | 53.8 (57) | 21.418 (<0.001) |  |  |  | 13.067 (<0.001) | 2.624 (0.105) | 3.238 (0.072) |
|  | No | 90.9 (10) | 56.4 (88) | 34.5 (41) | 46.2 (49) |  |  |  |  |  |  |  |
| Decline in mobility | Yes | 36.4 (4) | 68.9 (106) | 68.1 (81) | 70.8 (75) | 5.413 (0.144) |  |  |  |  |  |  |
|  | No | 63.6 (7) | 32.1 (50) | 31.9 (38) | 29.3 (31) |  |  |  |  |  |  |  |
| Moving into nursing home | Yes | 0.0 (0) | 38.5 (60) | 58.0 (119) | 50.0 (53) | 20.446 (<0.001) |  |  |  | 10.330 (0.001) | 3.426 (0.064) | 1.439 (0.230) |
|  | No | 100.0 (11) | 61.5 (96) | 42.0 (50) | 50.0 (53) |  |  |  |  |  |  |  |
| Low income | Yes | 9.1 (1) | 10.3 (16) | 10.9 (13) | 19.8 (21) | 5.984 (0.112) |  |  |  |  |  |  |
|  | No | 90.9 (10) | 89.7 (140) | 89.1 (85) | 80.2 (85) |  |  |  |  |  |  |  |
| Lack of support | Yes | 27.3 (3) | 44.9 (70) | 71.4 (85) | 76.4 (81) | 38.334 (<0.001) |  |  |  | 19.357 (<0.001) | 25.719 (<0.001) | 0.721 (0.396) |
|  | No | 72.7 (8) | 55.1 (86) | 28.6 (34) | 23.6 (25) |  |  |  |  |  |  |  |
| Others not informed about disease | Yes | 9.1 (1) | 47.4 (74) | 69.8 (83) | 74.5 (79) | 36.281 (<0.001) |  |  |  | 13.718 (<0.001) | 19.068 (<0.001) | 0.636 (0.425) |
|  | No | 90.9 (10) | 52.6 (82) | 30.3 (36) | 25.5 (27) |  |  |  |  |  |  |  |
| Others withdraw due to lack of time or unwillingness to invest time | Yes | 27.3 (3) | 55.8 (87) | 65.6 (78) | 53.8 (57) | 8.052 (0.045) |  |  |  | 2.689 (0.101) | 0.102 (0.750) | 3.238 (0.072) |
|  | No | 72.7 (8) | 44.2 (69) | 34.5 (41) | 46.2 (49) |  |  |  |  |  |  |  |
| Non-dementia-related impairments (e.g. poor hearing) | Yes | 0.0 (0) | 55.6 (5) | 0.0 (0) | 25.0 (1) | 4.625 (0.201) |  |  |  |  |  |  |
|  | No | 100.0 (2) | 44.4 (4) | 100.0 (3) | 75.0 (3) |  |  |  |  |  |  |  |
| Excessive demands in social contacts | Yes | 0.0 (0) | 22.2 (2) | 66.7 (2) | 50.0 (2) | 3.500 (0.321) |  |  |  |  |  |  |
|  | No | 100.0 (2) | 77.8 (7) | 33.3 (1) | 50.0 (2) |  |  |  |  |  |  |  |
| **Activation in an advanced dementia stage** |  |  |  |  |  |  |  |  |  |  |  |  |
| Touch | Yes |  | 66.7 (98) | 81.2 (82) | 77.6 (76) | 7.461 (0.024) |  |  |  | 6.344 (0.012) | 3.385 (0.066) | 0.402 (0.526) |
|  | No |  | 33.3 (49) | 18.8 (19) | 22.5 (22) |  |  |  |  |  |  |  |
| Singing or humming | Yes |  | 61.2 (90) | 88.1 (89) | 91.8 (90) | 40.718 (<0.001) |  |  |  | 21.563 (<0.001) | 28.269 (<0.001) | 0.761 (0.383) |
|  | No |  | 38.8 (57) | 11.9 (12) | 8.2 (8) |  |  |  |  |  |  |  |
| Moving together | Yes |  | 65.3 (96) | 69.3 (70) | 74.5 (98) | 2.325 (0.313) |  |  |  |  |  |  |
|  | No |  | 34.7 (51) | 30.7 (31) | 25.5 (25) |  |  |  |  |  |  |  |
| Holding hands | Yes |  | 61.2 (90) | 66.3 (67) | 64.3 (63) | 0.705 (0.703) |  |  |  |  |  |  |
|  | No |  | 38.8 (57) | 33.7 (34) | 35.7 (35) |  |  |  |  |  |  |  |
| Mirroring gestures, sounds or posture | Yes |  | 22.5 (33) | 47.5 (48) | 37.8 (37) | 17.562 (<0.001) |  |  |  | 17.116 (<0.001) | 6.750 (0.009) | 1.940 (0.164) |
|  | No |  | 77.6 (114) | 52.5 (53) | 62.2 (61) |  |  |  |  |  |  |  |
| Using familiar objects | Yes |  | 43.5 (64) | 59.4 (60) | 61.2 (60) | 9.609 (0.008) |  |  |  | 6.030 (0.014) | 7.359 (0.007) | 0.069 (0.793) |
|  | No |  | 56.5 (83) | 40.6 (41) | 38.8 (38) |  |  |  |  |  |  |  |
| Stimulating senses | Yes |  | 1.4 (2) | 2.0 (2) | 3.1 (3) | 0.859 (0.651) |  |  |  |  |  |  |
|  | No |  | 98.6 (145) | 98.0 (99) | 96.9 (95) |  |  |  |  |  |  |  |
| **Relationships that break off** |  |  |  |  |  |  |  |  |  |  |  |  |
| Friends and acquaintances |  |  | 52.7 (79) | 43.9 (50) | 49.5 (51) | 32.634 (0.173) |  |  |  |  |  |  |
| Contacts from work/school |  |  | 22.7 (34) | 23.7 (27) | 33.0 (34) |  |  |  |  |  |  |  |
| Contacts from social media |  |  | 5.33 (8) | 14.0 (16) | 10.7 (11) |  |  |  |  |  |  |  |
| Neighbors |  |  | 8.0 (12) | 8.8 (10) | 3.9 (4) |  |  |  |  |  |  |  |
| Family members |  |  | 3.3 (5) | 2.6 (3) | 1.0 (1) |  |  |  |  |  |  |  |
| Service providers |  |  | 2.7 (4) | 1.8 (2) | 1.0 (1) |  |  |  |  |  |  |  |
| **New social contacts** |  |  |  |  |  |  |  |  |  |  |  |  |
| Yes |  | 41.7 (5) | 45.5 (70) | 56.4 (62) | 43.8 (42) | 32.866 (0.005) |  |  |  |  |  |  |
| No |  | 41.7 (5) | 46.1 (71) | 28.2 (31) | 35.4 (34) |  |  |  |  |  |  |  |
| Rarely |  | 0.0 (0) | 1.3 (2) | 7.3 (8) | 7.3 (7) |  |  |  |  |  |  |  |
| **Triggering positive emotions** |  |  |  |  |  |  |  |  |  |  |  |  |
| Cuddling, physical closeness, or sex | Yes | 38.5 (5) | 47.7 (71) | 57.9 (62) | 48.5 (48) | 3.770 (0.287) |  |  |  |  |  |  |
|  | No | 61.5 (8) | 52.4 (78) | 42.1 (45) | 51.5 (51) |  |  |  |  |  |  |  |
|  |  |  |  |  |  |  |  |  |  |  |  |  |
| Conversations | Yes | 84.6 (11) | 53.0 (79) | 56.1 (60) | 38.4 (38) | 13.530 (0.004) |  |  |  | 0.234 (0.628) | 5.113 (0.024) | 6.453 (0.011) |
|  | No | 15.4 (2) | 47.0 (70) | 43.9 (47) | 61.6 (61) |  |  |  |  |  |  |  |
| Being together | Yes | 76.9 (10) | 92.0 (137) | 82.2 (88) | 81.8 (81) | 7.891 (0.048) |  |  |  | 5.509 (0.019) | 5.738 (0.017) | 0.006 (0.937) |
|  | No | 23.1 (3) | 8.1 (12) | 17.8 (19) | 18.2 (18) |  |  |  |  |  |  |  |
| Participating in daily activities | Yes | 38.5 (5) | 55.0 (82) | 78.5 (84) | 67.7 (67) | 19.320 (<0.001) | 1.321 (0.250) | 9.701 (0.002) | 4.272 (0.039) | 15.050 (<0.001) | 3.964 (0.046) | 3.081 (0.079) |
|  | No | 61.5 (8) | 45.0 (67) | 21.5 (23) | 32.3 (32) |  |  |  |  |  |  |  |
| Laughing together | Yes | 61.5 (8) | 76.5 (114) | 84.1 (90) | 85.9 (85) | 7.237 (0.065) |  |  |  |  |  |  |
|  | No | 38.5 (5) | 23.5 (35) | 15.9 (17) | 14.1 (14) |  |  |  |  |  |  |  |
| Joint activities (e.g. singing, travelling, playing games) | Yes | 76.9 (10) | 73.8 (110) | 87.9 (94) | 83.8 (83) | 8.781 (0.032) |  |  |  | 7.568 (0.006) | 3.455 (0.063) | 0.684 (0.408) |
|  | No | 23.1 (3) | 26.2 (39) | 12.2 (13) | 16.2 (16) |  |  |  |  |  |  |  |
| Biography work | Yes | 0.0 (0) | 26.9 (40) | 57.0 (61) | 57.6 (  57) | 42.836 (<0.001) |  |  |  | 23.721 (<0.001) | 23.586 (<0.001) | 0.007 (0.935) |
|  | No | 100.0 (13) | 73.2 (109) | 43.0 (46) | 42.4 (42) |  |  |  |  |  |  |  |
| Performing personal hygiene | Yes | 23.1 (3) | 16.8 (25) | 17.8 (19) | 12.1 (12) | 1.902 (0.593) |  |  |  |  |  |  |
|  | No | 76.9 (10) | 83.2 (124) | 82.2 (88) | 87.9 (87) |  |  |  |  |  |  |  |
| Visiting friends | Yes | 38.5 (5) | 26.9 (40) | 33.6 (36) | 29.3 (29) | 1.851 (0.604) |  |  |  |  |  |  |
|  | No | 61.5 (8) | 73.2 (109) | 66.4 (71) | 70.7 (70) |  |  |  |  |  |  |  |
| Interactions in nature with/(out) animals | Yes | 100.0 (1) | 50.0 (3) | 0.0 (0) | 50.0 (1) | 2.000 (0.572) |  |  |  |  |  |  |
|  | No | 0.0 (0) | 50.0 (3) | 100.0 (1) | 50.0 (1) |  |  |  |  |  |  |  |
| **Feeling needed** |  |  |  |  |  |  |  |  |  |  |  |  |
| Being involved in daily activities | Yes | 54.6 (6) | 83.9 (125) | 93.5 (100) | 92.9 (92) | 19.670 (<0.001) | 5.945 (0.015) | 16.533 (<0.001) | 15.008 (<0.001) | 5.354 (0.021) | 4.441 (0.035) | 0.023 (0.880) |
|  | No | 45.5 (5) | 16.1 (24) | 6.5 (7) | 7.1 (7) |  |  |  |  |  |  |  |
| Being asked for advice/own opinion | Yes | 72.7 (8) | 55.7 (83) | 51.4 (55) | 55.6 (55) | 1.997 (0.573) |  |  |  |  |  |  |
|  | No | 27.3 (3) | 44.3 (66) | 48.6 (52) | 44.4 (44) |  |  |  |  |  |  |  |
| Being asked for help | Yes | 63.6 (7) | 64.4 (96) | 65.4 (70) | 72.7 (72) | 2.089 (0.554) |  |  |  |  |  |  |
|  | No | 36.4 (4) | 35.6 (53) | 34.6 (37) | 27.3 (27) |  |  |  |  |  |  |  |
| When their motivation and effort is valued | Yes | 36.4 (4) | 42.3 (63) | 52.3 (56) | 49.5 (49) | 3.302 (0.347) |  |  |  |  |  |  |
|  | No | 63.6 (7) | 57.7 (86) | 47.7 (51) | 50.5 (50) |  |  |  |  |  |  |  |
| Assisting in activities they used to perform | Yes | 18.2 (2) | 61.1 (91) | 75.7 (81) | 77.8 (77) | 23.267 (<0.001) |  |  |  | 6.044 (0.014) | 7.595 (0.006) | 0.124 (0.725) |
|  | No | 81.8 (9) | 38.9 (58) | 24.3 (26) | 22.2 (22) |  |  |  |  |  |  |  |
| Receiving invitations to social activities | Yes | 36.4 (4) | 40.3 (60) | 53.3 (47) | 53.5 (53) | 6.546 (0.088) |  |  |  |  |  |  |
|  | No | 63.6 (7) | 59.7 (89) | 46.7 (50) | 46.5 (46) |  |  |  |  |  |  |  |
| Receiving visits | Yes | 27.3 (3) | 44.3 (66) | 33.6 (36) | 32.3 (32) | 5.240 (0.155) |  |  |  |  |  |  |
|  | No | 72.7 (8) | 55.7 (83) | 66.4 (71) | 67.7 (67) |  |  |  |  |  |  |  |
| **Promoting self-esteem** |  |  |  |  |  |  |  |  |  |  |  |  |
| Conveying appreciation | Yes | 75.0 (3) | 18.2 (18) | 15.5 (11) | 17.7 (12) | 9.039 (0.029) |  |  |  | 0.211 (0.646) | 0.008 (0.930) | 0.117 (0.733) |
|  | No | 25.0 (1) | 81.8 (81) | 84.5 (60) | 82.4 (56) |  |  |  |  |  |  |  |
| Carrying out meaningful activities | Yes | 0.0 (0) | 40.4 (40) | 62.0 (44) | 57.4 (39) | 13.125 (0.004) |  |  |  | 7.694 (0.006) | 4.646 (0.031) | 0.308 (0.579) |
|  | No | 100.0 (4) | 59.6 (59) | 38.0 (27) | 42.7 (29) |  |  |  |  |  |  |  |
| Engagement in social activities | Yes | 25.0 (1) | 27.3 (27) | 11.0 (8) | 23.5 (16) | 6.995 (0.072) |  |  |  |  |  |  |
|  | No | 75.0 (3) | 72.7 (72) | 89.0 (65) | 76.5 (52) |  |  |  |  |  |  |  |
| Experiencing competence | Yes | 33.3 (1) | 35.4 (35) | 30.1 (22) | 33.8 (23) | 0.525 (0.913) |  |  |  |  |  |  |
|  | No | 66.7 (2) | 64.7 (64) | 69.9 (51) | 66.2 (45) |  |  |  |  |  |  |  |
| **Providing safety and security** |  |  |  |  |  |  |  |  |  |  |  |  |
| Everyday support | Yes |  | 55.0 (82) | 45.8 (49) | 52.5 (52) | 2.181 (0.336) |  |  |  |  |  |  |
|  | No |  | 45.0 (67) | 54.2 (58) | 47.5 (47) |  |  |  |  |  |  |  |
| Performing personal hygiene | Yes |  | 19.5 (29) | 10.3 (11) | 5.1 (5) | 11.956 (0.003) |  |  |  | 3.983 (0.046) | 10.444 (0.001) | 1.963 (0.161) |
|  | No |  | 80.5 (120) | 89.7 (96) | 95.0 (94) |  |  |  |  |  |  |  |
| Conversations | Yes |  | 57.7 (86) | 43.9 (47) | 51.5 (51) | 4.751 (0.093) |  |  |  |  |  |  |
|  | No |  | 42.3 (63) | 56.1 (60) | 48.5 (48) |  |  |  |  |  |  |  |
| Hugging | Yes |  | 71.1 (106) | 67.3 (72) | 61.6 (61) | 2.453 (0.293) |  |  |  |  |  |  |
|  | No |  | 28.9 (43) | 32.7 (35) | 38.4 (38) |  |  |  |  |  |  |  |
| Playing together | Yes |  | 42.3 (63) | 43.9 (47) | 43.4 (43) | 0.075 (0.963) |  |  |  |  |  |  |
|  | No |  | 57.7 (86) | 56.1 (60) | 56.6 (56) |  |  |  |  |  |  |  |
| Singing | Yes |  | 50.3 (75) | 78.5 (84) | 68.7 (84) | 22.773 (<0.001) |  |  |  | 20.998 (<0.001) | 8.205 (0.004) | 2.563 (0.109) |
|  | No |  | 49.7 (74) | 21.5 (23) | 31.3 (31) |  |  |  |  |  |  |  |
| Eating together | Yes |  | 72.5 (108) | 66.4 (71) | 61.6 (61) | 3.312 (0.190) |  |  |  |  |  |  |
|  | No |  | 27.5 (41) | 33.6 (36) | 38.4 (38) |  |  |  |  |  |  |  |
| Sitting together (e.g. watching movies) | Yes |  | 47.0 (70) | 41.1 (44) | 47.5 (47) | 1.112 (0.574) |  |  |  |  |  |  |
|  | No |  | 53.0 (79) | 58.9 (63) | 52.5 (52) |  |  |  |  |  |  |  |
| Being active together | Yes |  | 49.0 (73) | 57.9 (62) | 66.7 (66) | 7.673 (0.022) |  |  |  | 2.002 (0.157) | 7.542 (0.006) | 1.663 (0.197) |
|  | No |  | 51.0 (76) | 42.1 (45) | 33.3 (33) |  |  |  |  |  |  |  |
| Family moments | Yes |  | 50.0 (1) | 33.3 (2) | 100.0 (2) | 2.667 (0.264) |  |  |  |  |  |  |
|  | No |  | 50.0 (1) | 66.7 (4) | 0.0 (0) |  |  |  |  |  |  |  |
| **Calm aggressive behavior** |  |  |  |  |  |  |  |  |  |  |  |  |
| Signaling that their feelings are taken seriously | Yes |  | 56.9 (83) | 92.1 (93) | 88.8 (87) | 52.782 (<0.001) |  |  |  | 36.176 (<0.001) | 28.287 (<0.001) | 0.629 (0.428) |
|  | No |  | 43.2 (63) | 7.9 (8) | 11.2 (11) |  |  |  |  |  |  |  |
| Distraction | Yes |  | 50.0 (73) | 53.5 (54) | 57.1 (56) | 1.211 (0.546) |  |  |  |  |  |  |
|  | No |  | 50.0 (73) | 46.5 (47) | 42.9 (42) |  |  |  |  |  |  |  |
| Figuring out underlying reasons | Yes |  | 16.4 (24) | 35.6 (36) | 19.4 (19) | 13.428 (0.001) |  |  |  | 11.973 (0.001) | 0.351 (0.553) | 6.572 (0.010) |
|  | No |  | 83.6 (122) | 64.4 (65) | 80.6 (79) |  |  |  |  |  |  |  |
| Staying calm | Yes |  | 80.8 (118) | 85.2 (86) | 83.7 (82) | 0.846 (0.655) |  |  |  |  |  |  |
|  | No |  | 19.2 (28) | 14.9 (15) | 16.3 (16) |  |  |  |  |  |  |  |
| Touch | Yes |  | 53.4 (78) | 66.3 (67) | 48.0 (47) | 7.315 (0.026) |  |  |  | 4.106 (0.043) | 0.701 (0.402) | 6.865 (0.009) |
|  | No |  | 46.6 (68) | 33.7 (34) | 52.0 (51) |  |  |  |  |  |  |  |
| Leaving the situation | Yes |  | 24.7 (36) | 13.9 (14) | 21.4 (21) | 4.318 (0.115) |  |  |  |  |  |  |
|  | No |  | 75.3 (110) | 86.1 (87) | 78.6 (77) |  |  |  |  |  |  |  |
| Individual strategies (e.g. saying stop) | Yes |  | 2.1 (3) | 5.0 (5) | 1.0 (1) | 3.330 (0.189) |  |  |  |  |  |  |
|  | No |  | 98.0 (143) | 95.1 (96) | 99.0 (97) |  |  |  |  |  |  |  |
| **Influence depressive symptoms** |  |  |  |  |  |  |  |  |  |  |  |  |
| Signaling that their feelings are taken seriously | Yes |  | 55.8 (82) | 72.3 (73) | 82.7 (81) | 20.668 (<0.001) |  |  |  | 6.950 (0.008) | 19.066 (<0.001) | 3.060 (0.080) |
|  | No |  | 44.2 (65) | 27.7 (28) | 17.4 (17) |  |  |  |  |  |  |  |
| Distraction | Yes |  | 47.6 (70) | 36.6 (37) | 34.7 (34) | 5.070 (0.079) |  |  |  |  |  |  |
|  | No |  | 52.4 (77) | 63.4 (64) | 65.3 (64) |  |  |  |  |  |  |  |
| Figuring out underlying reasons | Yes |  | 27.9 (41) | 47.5 (48) | 31.6 (31) | 10.748 (0.005) |  |  |  | 10.030 (0.002) | 0.397 (0.529) | 5.248 (0.022) |
|  | No |  | 72.1 (106) | 52.5 (53) | 68.4 (67) |  |  |  |  |  |  |  |
| Being involved in daily activities | Yes |  | 67.4 (99) | 79.2 (80) | 76.5 (75) | 4.997 (0.082) |  |  |  |  |  |  |
|  | No |  | 32.7 (48) | 20.8 (21) | 23.5 (23) |  |  |  |  |  |  |  |
| Touch | Yes |  | 65.3 (96) | 70.3 (71) | 68.4 (67) | 0.715 (0.699) |  |  |  |  |  |  |
|  | No |  | 34.7 (51) | 28.7 (30) | 31.6 (31) |  |  |  |  |  |  |  |
| Being involved in interest-based activities | Yes |  | 42.2 (62) | 54.5 (55) | 66.3 (65) | 13.951 (0.001) |  |  |  | 3.622 (0.057) | 13.736 (<0.001) | 2.928 (0.087) |
|  | No |  | 57.8 (85) | 45.5 (46) | 33.7 (33) |  |  |  |  |  |  |  |

Notes: *, only if overall comparison was p<0.05; §, not reported if n<5; FC, family caregiver; n, number of participants; p, level of significance; PC, professional caregiver; PWD, people with dementia; x2, chi square test.

**Supplementary table S3.** Differences in Likert scale answers between PWD, family caregiver (FC), professional caregiver (PC), and people otherwise connected to dementia care; n=476

| Question | Overall comparison | | | | | | | | | Pairwise comparison* | | | | | |
| --- | --- | --- | --- | --- | --- | --- | --- | --- | --- | --- | --- | --- | --- | --- | --- |
|  | PWD (n=14) | | FC (n=182) | | PC (n=145) | | Others (n=135) | |  | PWD - FC | PWD - PC | PWD - Others | FC - PC | FC - Others | PC - Others |
|  | n | M±  SD | n | M±  SD | n | M±  SD | n | M±  SD | X²  (p) | M∆, p | M∆, p | M∆, p | M∆, p | M∆, p | M∆, p |
| **Ranking activities for the initiation of social interactions^1^** |  |  |  |  |  |  |  |  |  |  |  |  |  |  |  |
| Organized activities | 7 | 2.7±2.4 | 162 | 3.1±2.1 | 139 | 2.5±1.9 | 127 | 3.2±2.1 | 11.148 (0.011) | 0.4, 0.629 | 0.3, 0.740 | 0.5, 0.554 | 0.6, 0.007 | 0.1, 0.713 | 0.7, 0.004 |
| Activities from religious institutions | 4 | 7.8±0.5 | 148 | 6.1±2.0 | 131 | 5.0±2.0 | 117 | 5.8±1.8 | 28.412 (<0.001) | 1.7, 0.089 | 2.8, 0.005 | 2.0, 0.046 | 1.1, <0.001 | 0.3, 0.218 | 0.8, 0.001 |
| Doctor’s appointment or therapy | 9 | 3.1±1.9 | 163 | 4.2±1.9 | 131 | 4.8±1.7 | 121 | 2.1±4.1 | 13.501 (0.004) | 1.1, 0.097 | 1.7, 0.009 | 1.0, 0.127 | 0.6, 0.005 | 0.1, 0.726 | 0.7, 0.003 |
| Support services | 7 | 3.0±2.5 | 166 | 3.0±1.9 | 137 | 3.0±1.9 | 124 | 3.3±1.9 | 2.984 (0.394) |  |  |  |  |  |  |
| Procurement of food and medicine | 8 | 4.1±2.1 | 150 | 4.7±2.0 | 129 | 5.3±1.8 | 120 | 4.6±1.9 | 8.609 (0.035) | 0.6, 0.374 | 1.1, 0.107 | 0.5, 0.488 | 0.5, 0.027 | 0.1, 0.573 | 0.6, 0.008 |
| Leisure activities | 10 | 2.6±1.8 | 162 | 3.5±1.8 | 135 | 3.8±1.9 | 125 | 4.3±2.0 | 13.360 (0.004) | 0.9, 0.128 | 1.2, 0.047 | 1.7, 0.008 | 0.3, 0.184 | 0.7, 0.001 | 0.4, 0.069 |
| Encounters in the neighborhood | 10 | 3.2±1.6 | 163 | 3.7±1.9 | 132 | 4.1±1.9 | 125 | 3.3±1.9 | 11.320 (0.010) | 0.5, 0.377 | 0.9, 0.143 | 0.1, 0.836 | 0.4, 0.100 | 0.4, 0.065 | 0.8, 0.001 |
| Coincidentally | 5 | 4.2±2.3 | 147 | 6.7±1.8 | 127 | 7.2±1.5 | 120 | 7.0±1.7 | 19.721 (<0.001) | 2.5, 0.001 | 3.0, <0.001 | 2.8, <0.001 | 0.6, 0.005 | 0.4, 0.074 | 0.2, 0.332 |
| **Frequency of social interactions^2^** |  |  |  |  |  |  |  |  |  |  |  |  |  |  |  |
| Conduct active conversation | 13 | 3.4±1.3 | 175 | 2.8±0.8 | 136 | 3.0±0.7 | 126 | 2.7±0.7 | 12.939 (0.005) | 0.6, 0.006 | 0.4, 0.089 | 0.7, 0.003 | 0.2, 0.011 | 0.1, 0.474 | 0.3, 0.002 |
| Experience touch | 13 | 3.1±1.3 | 174 | 3.2±0.9 | 135 | 3.3±0.8 | 124 | 2.9±0.8 | 10.993 (0.012) | 0.1, 0.619 | 0.2, 0.418 | 0.1, 0.577 | 0.1, 0.421 | 0.3, 0.010 | 0.3, 0.001 |
| Have sexual intercourse | 10 | 1.0±0.0 | 158 | 1.5±0.7 | 124 | 2.2±0.7 | 108 | 2.1±0.6 | 71.688 (<0.001) | 0.5, 0.015 | 1.2, <0.001 | 1.1, <0.001 | 0.6, <0.001 | 0.5, <0.001 | 0.1, 0.362 |
| **Importance of social interactions^3^** |  |  |  |  |  |  |  |  |  |  |  |  |  |  |  |
| Regular social interactions preserve PWDs’ cognitive performance | 11 | 3.3±1.6 | 147 | 4.0±1.0 | 99 | 4.3±0.8 | 96 | 4.4±0.8 | 13.021 (0.005) | 0.7, 0.017 | 1.0, 0.001 | 1.1, <0.001 | 0.3, 0.007 | 0.4, 0.001 | 0.1, 0.487 |
| Involving PWD in daily activities is important to reduce dementia symptoms | 10 | 3.5±1.4 | 147 | 3.9±1.0 | 99 | 4.0±1.0 | 96 | 4.1±0.8 | 2.974 (0.396) |  |  |  |  |  |  |
| Socially integrated PWD have a high quality of life | 12 | 3.8±1.3 | 146 | 4.0±1.1 | 99 | 4.3±0.8 | 97 | 4.4±0.7 | 13.186 (0.004) | 0.1, 0.670 | 0.5, 0.085 | 0.6, 0.039 | 0.4, 0.002 | 0.5, <0.001 | 0.1, 0.454 |
| **Methods to increase social interactions** |  |  |  |  |  |  |  |  |  |  |  |  |  |  |  |
| Social interactions are actively established^4^ | 11 | 3.2±1.7 | 152 | 4.4±0.9 | 113 | 4.5±0.8 | 105 | 4.3±0.8 | 6.619 (0.085) |  |  |  |  |  |  |
| Fixed social professional contact person^4^ | 11 | 3.1±1.8 | 152 | 4.3±1.1 | 113 | 4.4±0.9 | 105 | 4.5±0.8 | 6.900 (0.075) |  |  |  |  |  |  |
| Showing understanding for dementia symptoms^4^ | 12 | 4.1±1.4 | 154 | 4.7±0.7 | 114 | 4.8±0.5 | 105 | 4.9±0.4 | 5.021 (0.172) |  |  |  |  |  |  |
| Offers from the community^4^ | 12 | 2.3±1.4 | 148 | 4.1±1.1 | 115 | 4.2±1.1 | 106 | 4.4±0.9 | 20.525 (<0.001) | 1.8, <0.001 | 1.9, <0.001 | 2.0, <0.001 | 0.1, 0.386 | 0.3, 0.029 | 0.2, 0.206 |
| Offers from associations^4^ | 12 | 2.4±1.4 | 147 | 3.9±1.1 | 113 | 4.1±1.0 | 106 | 4.4±0.8 | 25.675 (<0.001) | 1.5, <0.001 | 1.7, <0.001 | 2.0, <0.001 | 0.2, 0.175 | 0.5, <0.001 | 0.3, 0.016 |
| Offers from religious institutions^4^ | 12 | 1.2±0.4 | 144 | 3.3±1.3 | 115 | 4.0±1.1 | 105 | 3.8±1.0 | 46.244 (<0.001) | 2.1, <0.001 | 2.9, <0.001 | 2.7, <0.001 | 0.7, <0.001, | 0.5, <0.001 | 0.1, 0.379 |
| Exchange between relatives and professionals^4^ | 11 | 3.2±1.9 | 153 | 4.60±0.7 | 114 | 4.8±0.5 | 105 | 4.7±0.6 | 7.535 (0.056) |  |  |  |  |  |  |
| Technology-supported interventions (e.g. apps) could support the social interaction^3^ | 10 | 2.4±1.6 | 140 | 2.0±1.1 | 97 | 2.4±1.1 | 94 | 2.6±0.9 | 18.332 (<0.001) | 0.4, 0.291 | 0.0, 0.995 | 0.2, 0.591 | 0.4, 0.009 | 0.6, <0.001 | 0.2, 0.222 |
| Robots (e.g. in the form of an animal or human) could support the social interaction^3^ |  |  | 139 | 2.3±1.2 | 98 | 2.5±1.2 | 95 | 2.7±1.1 | 6.618 (0.037) |  |  |  | 0.2, 0.264 | 0.4, 0.007 | 0.3, 0.145 |

Notes: *, only if overall comparison was p<0.05; FC, family caregiver; M, mean; M∆, difference in mean; n, number of participants; p, level of significance; PC, professional caregiver; PWD, people with dementia; SD, standard deviation; x2, chi square as of Kruskall-Wallis test; 1, rank in order of importance with 1 indicating the most important; 2, Likert scale level of frequency with never (1) to always (5); 3, Likert scale level of agreeing with not very (1) to strongly (5); 4, Likert scale level of importance with not very (1) to very (5).
